# Comparing Sanitation Delivery Modalities in Urban Informal Settlement Schools: A Randomized Trial in Nairobi, Kenya

**DOI:** 10.3390/ijerph13121189

**Published:** 2016-11-30

**Authors:** Kate Bohnert, Anna N. Chard, Alex Mwaki, Amy E. Kirby, Richard Muga, Corey L. Nagel, Evan A. Thomas, Matthew C. Freeman

**Affiliations:** 1 Department of Environmental Health, Emory University, Atlanta, GA 30322, USA; kate.bohnert@gmail.com (K.B.); achard@emory.edu (A.N.C.); 2 Cooperative for Assistance and Relief Everywhere (CARE) Kenya, Muchai Road, P.O. Box 43864, Nairobi 00100, Kenya; alex.mwaki@gmail.com; 3 Hubert Department of Global Health, Emory University, Atlanta, GA 30322, USA; aekirby@emory.edu; 4 Department of Health Sciences, Great Lakes University of Kisumu, P.O. Box 2224, Kisumu 40100, Kenya; drmuga@yahoo.com; 5 School of Public Health, Oregon Health and Science University, Portland State University, Portland, OR 97239, USA; nagelc@ohsu.edu; 6 College of Engineering and Computer Science, Portland State University, Portland, OR 97201, USA; evthomas@pdx.edu

**Keywords:** sanitation, school, informal settlements, sanitation service delivery, private sector provision

## Abstract

The provision of safely managed sanitation in informal settlements is a challenge, especially in schools that require durable, clean, sex-segregated facilities for a large number of children. In informal settlements in Nairobi, school sanitation facilities demand considerable capital costs, yet are prone to breakage and often unhygienic. The private sector may be able to provide quality facilities and services to schools at lower costs as an alternative to the sanitation that is traditionally provided by the government. We conducted a randomized trial comparing private sector service delivery (PSSD) of urine-diverting dry latrines with routine waste collection and maintenance and government standard delivery (GSD) of cistern-flush toilets or ventilated improved pit latrines. The primary outcomes were facility maintenance, use, exposure to fecal contamination, and cost. Schools were followed for one school year. There were few differences in maintenance and pathogen exposure between PSSD and GSD toilets. Use of the PSSD toilets was 128% higher than GSD toilets, as measured with electronic motion detectors. The initial cost of private sector service delivery was USD 2053 (KES 210,000) per school, which was lower than the average cost of rehabilitating the government standard flush-type toilets (USD 9306 (KES 922,638)) and constructing new facilities (USD 114,889 (KES 1,169,668)). The private sector delivery of dry sanitation provided a feasible alternative to the delivery of sewage sanitation in Nairobi informal settlements and might elsewhere in sub-Saharan Africa.

## 1. Introduction

More than 800 million people live in urban informal settlements, a number that is expected to double by 2030 [1]. This rapid increase in urbanization has led to overcrowding and limited access to water and sanitation [2]. Technologies have been developed to meet the basic sanitation needs of those living in informal settlements. Pit latrines, which collect waste in pits in the ground, and cistern-flush toilets, which utilize simplified sewage similar to conventional sewage but with pipes buried at a shallower depth in a smaller piped network, provide alternatives to flying toilets and open defecation [3,4]. However, these sanitation approaches are met with challenges, including limited investment in sanitation, inappropriate technology design, sustainability, and a paucity of administrative oversight for service provision in these marginalized areas [5,6]. Because of ineffective sanitation service delivery, residents of informal settlements are increasingly exposed to sanitation-related diseases due to contact with poorly managed human excreta [3].

For the millions of individuals living in informal settlements in sub-Saharan Africa, inadequate funding for urban sanitation and a lack of sanitation regulations exacerbate poor sanitation provision. Over 65% of those living in informal settlements in Nairobi, Kenya, have inadequate sanitation [7]. As a result, residents of these informal settlements, especially children under five years of age, are disproportionately at risk for sanitation-related diseases compared to the rural population [8]. There is evidence that water, sanitation, and hygiene (WASH) interventions can improve health in children at home [9,10,11] and at school [12,13,14]. The provision of WASH services in schools, including WASH-related supplies and activities to promote continued access to safe and hygienic facilities, can improve the overall quality of primary schools and the pupils’ health [15,16,17,18].

Safely managing sanitation needs in informal urban settlements is challenging, especially in schools, where facilities must be sex-segregated, hygienic, durable, and used by a large number of students. Further, despite substantial capital costs, school sanitation facilities are prone to disrepair and are often not well maintained. Schools experience many of the same obstacles as communities in informal settlements when providing WASH services. Space is limited to build WASH facilities, and certain sanitation technologies can be inappropriate when schools have inconsistent access to water and/or sewage [15]. The combination of unstable soil formation and high water tables makes latrine construction expensive [19]. Collection and transport of fecal waste is difficult because of narrow and muddy roads [20]. Low funding and low prioritization of WASH in schools also hinders sanitation service delivery to schools [17]. These challenges suggest the need for alternative service delivery mechanisms to address the basic sanitation and hygiene needs of schoolchildren in informal settlements.

There is limited evidence on the effect of different sanitation and hygiene provision modalities to overcome the challenges among schools in informal settlements. In close collaboration with Ministry of Education, Science and Technology of Kenya and the Nairobi City Council, we conducted a randomized controlled trial comparing a private sector sanitation service delivery (PSSD) model of urine-diverting dry sanitation facilities with routine waste collection and maintenance to the government standard delivery (GSD) of ventilated improved pit latrines or sewered cistern-flush toilets in four informal settlements in Nairobi, Kenya. The study was conducted within the urban informal settlements in Nairobi City County, including the areas of Mukuru, Mathare, Kamukunji, and Makadara. The overall population of Nairobi City County is over three million inhabitants, and it is estimated that 60% of this population lives in informal settlements [21]. The primary schools in these informal settlements have inadequate sanitation facilities for both large and small populations. The current Government of Kenya (GoK) standard for school toilet:pupil ratios are 1:25 for girls and 1:35 for boys plus a urinal [22].

This study was part of SWASH+, an applied research project designed to address policy-relevant, school-based challenges and to provide evidence to ensure that decision-makers within the GoK have access to high quality data to inform investment decisions on the provision of school WASH services. SWASH+ has included several studies, including large randomized trials to assess the health and educational benefits and sustainability of school WASH to inform an increase in recurrent payments to improve school conditions [12,13,18,23], collected data on life-cycle costs for school WASH [24], and addressed issues of menstrual hygiene management to inform free provision of pads [25]. The purpose of this study was to assess the feasibility of a private sector, school-based sanitation and hygiene service delivery model and inform GoK policy and guidelines for provision of school sanitation and hygiene in Nairobi City County, Kenya. Feasibility was defined as the ability to provide sustained sanitation services that were similar or superior to standard government sanitation approaches. The primary outcomes were toilet maintenance, toilet use, exposure to fecal contamination, prevalence of handwashing after toilet use, and cost of PSSD and GSD facilities.

## 2. Materials and Methods

### 2.1. School Selection

Twenty schools were randomly selected from a list provided by the Nairobi City County. Sample size was determined by the maximum number of schools that could be included given the study budget. Primary schools were considered eligible for inclusion if they had poor sanitation conditions, the willingness to pay for a private sector sanitation service delivery model or receive government standard flush-type sanitation facilities, sufficient space to accommodate the new facilities, and an adequate population to support the use of five sanitation facilities. Selected schools were visited to confirm eligibility. Schools were then randomly allocated to one of two intervention arms (10 schools in each arm), stratifying the group by private (4 schools in each arm) and public (6 schools in each arm). Randomized selection and allocation was conducted using a random number generator in Microsoft Excel (Redmond, WA, USA) by Emory University research staff.

### 2.2. Intervention

Schools allocated to the PSSD arm received five Fresh Life Toilets (FLTs) per school and a hygiene curriculum to promote behavior change provided by Sanergy, a private sector enterprise based in Mukuru, Nairobi. Sanergy adapted their business model (a private franchise of prefabricated toilets operated by micro-entrepreneurs throughout Nairobi’s informal settlements) in order to provide FLTs, handwashing facilities, maintenance support, and a comprehensive hygiene promotion curriculum for schools in the informal settlements. The FLTs are urine-diverting dry toilets, which have cartridges that collect the waste. A waste collection team removes and replaces the cartridges to dispose of the waste on a daily basis. In collaboration with WASH United, a non-profit behavior change communication organization, Sanergy organized a two-day training of teachers, where Sanergy provided them with the knowledge, resources, and instructional tactics they needed to educate their pupils about WASH and to encourage adoption of healthy behaviors. Soap was not provided as part of the intervention.

The GSD arm received sanitation packages typical of GoK provision of school sanitation—five stances of cistern-flush toilets connected to the municipal septic system designed by an architect at Nairobi City County (NCC). Implementation of this arm by local contractors was facilitated by Cooperative for Assistance and Relief Everywhere (CARE), an international non-governmental organization (NGO) focused on serving individuals in the poorest communities in the world. These designs were derived from approved GoK blueprints and conformed to GoK policies. Some schools had toilet facilities that were in disrepair and consequently were not in use; rehabilitation of existing facilities was completed in four schools in the GSD arm. One school received five stances of ventilated improved pit (VIP) latrines rather than cistern-flush toilets due to lack of space to build a septic tank. A table comparing PSSD and GSD components can be found in Table 1.

**Figure 1:**
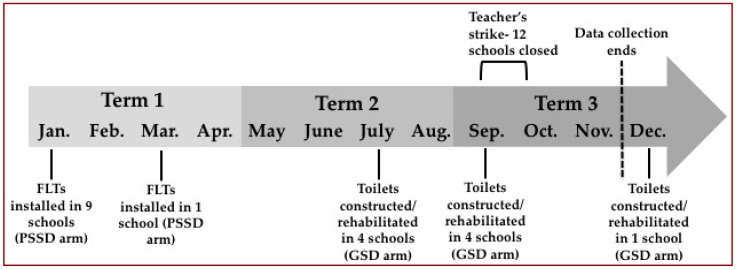
Timeline of study components.

Installation of FLTs in schools in the PSSD arm was completed by December 2014 (with the exception of one school, which was completed in March 2015). Eight of the ten GSD arm schools had completed construction by July 2015. The remaining two schools completed construction by November 2015. A timeline of intervention delivery is shown in Figure 1.

Since construction of the flush-toilets in the GSD arm was completed at different times in Term 2 (May–July), we opted to report data from Term 1 (January–March) and Term 3 (September–November) only. Given these construction delays in the GSD arm, data from Term 1 assess the impact of FLTs in the PSSD arm versus no new delivery in the GSD arm, whereas Term 3 compares the two different interventions.

### 2.3. Data Collection and Analysis

We collected data from January 2015 to November 2015. Each school was visited twice per term, for a total of six visits throughout the school year, with the exception of one school in the GSD arm, which was visited once in Term 1. All visits were unannounced. Two trained enumerators collected the following data at each visit: school-wide roll call, head teacher interview, structured facility observations, hand washing and toilet use observations, and environmental swabs or hand rinses.

One of the enumerators interviewed the head teacher or school director. The survey included questions about enrollment, activities influencing enrollment, the number and functionality of sanitation facilities, and maintenance of toilets. That enumerator also conducted visual inspections of drinking water containers, rainwater harvesting tanks, toilet compartments, and hand washing stations (if available). Observations were made for safety, privacy, functionality, and cleanliness of the sanitation facilities. The head teacher interview and structured facility observations were recorded in the Open Data Kit (ODK) mobile application [26].

Data on toilet maintenance was collected through the head teacher interview and structured facility observations. A scale score was constructed by assigning values to five binary (yes/no) toilet conditions observed during the structured facility observation: availability of cleaning materials, absence of flies, no odor, no visible feces, and absence of urine/stagnant water. Affirmative responses were assigned one point, and values were summed to create a scale of toilet maintenance ranging from 0 (very dirty) to 5 (very clean). In addition, head teachers reported the weekly frequency of toilet cleaning to quantify toilet maintenance.

We calculated toilet use using three measures: observed signs of use from the structured facility observation, direct observation of toilet use, and detected use from toilet sensors. For observed signs of use, the enumerator noted whether or not a toilet appeared to be in use. For direct observation, the enumerators were seated near the toilet area and recorded the number of pupils entering/exiting each toilet compartment or toilet block from 08:00 a.m. to 04:30 p.m. They also observed handwashing behaviors from where they were positioned. Handwashing behaviors of pupils utilizing the toilet were recorded as handwashing with water only, handwashing with soap or ash, or no handwashing. We deployed Passive Latrine Use Monitors (PLUMs) (SweetSense, Portland, OR, USA) in toilet blocks to measure usage in an unbiased manner [27]. In the case of the FLTs, the PLUM was placed directly over each single toilet stall. In the case of the cistern-flush (GSD) toilets, the PLUM was placed at the entrance and thereby captured movement into and out of the toilet blocks, which varied between 2 and 10 stalls. The PLUMs were rotated every three weeks between schools. The PLUMs measured use with an infrared motion detector that detected movement with a maximum resolution of 3 s per “click” of the motion detector. This raw movement data was aggregated into 24 hourly periods per day. The analysis period was constrained from 1 September to 30 November, covering the third academic term of the year, and the first and last days of sensor installation periods were not included in the analysis, to reduce potential reactivity.

Exposure to fecal contamination was measured using environmental swabs and hand rinses that were analyzed for *E. coli*, a commonly used fecal indicator bacteria [28]. At four visits, the enumerator collected composite swab samples from 10 randomly selected toilets in each school (800 samples total) using EnviroMax plus Sterile Environmental Sampling Swabs. The enumerator swabbed five square inches of the wall surface as well as the entirety of the interior door handle (when present) with the same swab. The wall surface area location swabbed was similar from toilet to toilet. In order to measure variations in *E. coli* exposure throughout the day in toilets, at the last visit we sampled six random toilets in each school at three fixed time points (09:00 a.m., 10:30 a.m., and 12:00 p.m.) in a day (360 samples total). Swabs were kept in a cooler with ice packs for a maximum of 6 h until they were transported to the lab, where they were processed immediately.

Enumerators collected hand rinses from 20 randomly selected pupils in each school at two different visits during Terms 2 and 3 (800 samples total). Each selected pupil placed his or her right hand in a 500-mL Whirl-Pak bag (Nasco, Fort Atkinson, WI, USA) containing 250 mL phosphate buffered saline (PBS) solution [29]. The enumerator then gently massaged the fingers and palm of the hand for 30 s to remove any particles. The pupil’s left hand was placed in the same bag and rinsed as described. The bag was closed, stored in a cooler with ice packs, and transported to the lab, where the samples were processed immediately.

The swabs and hand rinses were processed for *E. coli* using standard membrane filtration and m-ColiBlue24^®^ broth (Hach, Loveland, CO, USA) [30]. Seven mL of sterile phosphate buffered saline, pH 7.2 with 0.04% Tween-80 (PBST) was added to each swab container, vortexed for 30 s, incubated for 5 min, and then vortexed again for 30 s [31]. The swab elute was diluted in sterile PBS, processed by membrane filtration and plated on mColiBlue24 broth media [31]. Handrinses were tested for *E. coli* directly by membrane filtering 100 mL of the rinse solution without dilution and plating on mColiBlue24 broth media. Plates were incubated at 37.5 ± 0.5 °C. After 24 h, blue *E. coli* colonies were counted. The data are reported as colony-forming units (CFUs) per swab or hands, after adjusting for dilutions and filtered volume. Plates with ≥200 CFU were recorded as too numerous to count (TNTC) and were set to 200 CFU for the analyses. For handrinses, data are reported as presence of any *E. coli* on the pupils’ hands (<100 CFU/hands) versus high contamination of *E. coli* (≥100 CFU/hands). 

The cost of both interventions was tracked throughout the study. Cost in Kenyan shillings and person hours were recorded in an Excel spreadsheet and compared side by side. Costs were tracked by CARE and Sanergy for the two respective study arms using mutually agreed upon parameters and compiled into a table for comparison.

All data were cleaned and analyzed in Stata v12 (College Station, TX, USA). For each outcome, three comparisons were analyzed: (1) FLTs in the PSSD arm compared to existing toilets in the GSD arm in Term 1, representing a comparison of private sector service delivery versus no sanitation improvement; (2) FLTs in the PSSD arm compared to newly constructed facilities in the GSD arm in Term 3, representing private sector service delivery versus government standard sanitation provision; and (3) FLTs over all three terms. For comparisons 1 and 2, random effects linear (continuous outcomes) and logistic (binary outcomes) regression models were utilized with the data clustered at the school level to analyze differences in primary outcomes between interventions. For comparison 3, data were tested for trend across all three terms using the nptrend command.

Mixed-effects negative binomial regression was used to compare daily rates of use measured by the motion detector enabled sensor PLUMs between FLTs and government standard toilets. We fit a three-level, random intercept model to adjust for the clustering of days within toilets (level 2) and schools (level 3). The model contained an offset term in order to account for the number of individual toilets in the toilet block and robust standard errors were calculated for model coefficients [32]. The coefficient corresponding to the predicted difference between the groups was exponentiated to yield the ratio of use (rate ratio) in the FLTs over the government standard toilets. To assess for potential bias resulting from differences between toilets in the number of weekend days (when usage is presumably lower) that the sensors were installed, we conducted a sensitivity analysis modeling only rates of use during weekdays. We also conducted a sensitivity analysis in which install and removal days were retained in the analysis.

### 2.4. Ethics

The study was conducted in accordance with the Declaration of Helsinki. Ethics approval was granted by the Institutional Review Board at Emory University (IRB00079064, approved 24 January 2015) and the Ethical Review Committee at the Great Lakes University of Kisumu (GREC/191/01/2015, approved 6 January 2015). A waiver of parental consent was granted and head teachers of each school signed an in loco parentis form. For hand rinses, informed oral assent was obtained from all participants. The study adhered to CONSORT guidelines for randomized trials (Figure S1, Table S1).

## 3. Results

### 3.1. Maintenance and Cleanliness

Toilet maintenance characteristics are shown in Table 2. In Term 1, the FLTs in the PSSD arm were significantly cleaner (score 4.7/5.0) compared to the existing toilets in the GSD arm (score 2.6/5.0; *p* = 0.001). In Term 3, the FLTs were significantly cleaner (4.0) than the new toilets in the GSD arm (2.6; *p* = 0.002). However, the maintenance score for FLTs decreased each term, indicating decreased maintenance over time. There was no difference in the average number of days per week the FLTs were cleaned (5.0) compared to the existing toilets in the GSD arm (4.7) in Term 1 (*p* = 0.16). In Term 3, the average number of days for toilet cleaning was similar per week for the FLTs and the new toilets in the GSD arm (4.1 and 4.2, respectively, *p* = 0.99). There was no statistical difference in the average number of days that toilets were cleaned across terms for FLTs in the PSSD arm (5, 4.8, 4.1; *p* = 0.9). 

### 3.2. Access and Use of Sanitation Facilities

Toilet use characteristics are shown in Table 2. A similar percentage of FLTs (94%) were unlocked in Term 1 compared to the existing toilets in the GSD arm (84%; *p* = 0.37). Of these unlocked toilets, 93% of FLTs and 100% of existing GSD toilets appeared to be in use. Results were similar in Term 3 comparing FLTs to new toilets in the GSD arm. All of the unlocked FLTs and new GSD toilets appeared to be in use in Term 3. Use among FLTs in the PSSD arm across all three terms was similar.

The percentage of pupils that used an FLT in one day’s time in Term 1 was 12% compared to 14% of pupils using existing toilets in the GSD arm. The percentage of pupils that used an FLT in Term 3 was 15% compared to 20% of pupils using new toilets in the GSD arm. There was no statistical difference in FLT usage across terms in the PSSD arm (*p* = 0.5).

### 3.3. Handwashing

In Term 1, the percent of students observed handwashing with soap was higher in the PSSD arm compared to the GSD arm (*p* = 0.003), while the percent of students observed not washing their hands was higher in the GSD arm (*p* = 0.004). There was no difference in the percent of students who washed their hands with water only in Term 1 (*p* = 0.43). There were no differences in observed handwashing practices between the PSSD and GSD arms in Term 3. The percentage of students practicing handwashing with soap following FLT use was not different across all three terms (*p* = 0.54).

### 3.4. Sensor-Detected Use

Sensor data was available for a total of 25 toilets between 1 September and 30 November 2015. Of these, 17 were FLTs (PSSD arm) and eight were new cistern-flush toilets (GSD arm). All of the FLTs were individual units. In contrast, all of the cistern-flush toilets contained multiple individual toilets per toilet block (range 2–11). Not counting installation and removal days, the sensor was in place and functional for an average of 11.6 days (standard deviation (SD) = 4.4) in the FLTs and 10.4 days (SD = 5.7) in the GSD toilets. The proportion of these days that were weekdays was roughly equivalent in both groups (67.5% in the FLTs vs. 68.5% in the GSD toilets).

Because the PLUM installation locations varied between the PSSD arm and the GSD arm, structured observations combined with PLUM monitoring were conducted in both arms to estimate a scaling factor for estimating usage events to PLUM clicks—a recording of movement with a resolution of 3 s. A total of 1.741 unique observations at FLT locations corresponded to recorded PLUM data, with a median rate of 8.6 clicks per use. In the case of the GSD toilets, only 164 observations with corresponding PLUM data were available, indicating a median rate of 8.2 clicks per use. 

The mean number of clicks among individual toilets in the FLTs was 220.0 (SD = 177.5) and in the GSD toilets was 76.1 (SD = 84.7). The median click count was 247.0 (interquartile range (IQR) = 344.0) in the FLTs and 39.5 (IQR = 124.5) in the GSD toilets. These rates were scaled by the median clicks per use in each arm, identified through structured observations. In the case of the GSD toilets, the PLUM recorded events were then further scaled by the number of toilet stalls observed per PLUM.

Statistical analysis of sensor data revealed significantly higher daily use among the FLTs in the PSSD arm (RR = 2.28, 95% CI = 1.53–3.39, *p* < 0.001) when compared with cistern-flush toilets in the GSD arm. Comparable results were found in sensitivity analyses examining usage rates on weekdays only (RR = 2.41, 95% CI = 2.06–2.81, *p* < 0.001) or when recorded use on the days of sensor install and removal was included (RR = 2.16, 95% CI = 1.72–2.73, *p* < 0.001).

FLT use in Term 1 was 19.85 uses per toilet per day (SD = 23.21); in Term 2 was 31.79 (SD = 30.04) and in Term 3 was 25.52 (SD = 20.59). Applying an ANOVA adjusted for clustering, there was no difference in mean FLT use between the terms (*p* = 0.42).

### 3.5. Exposure

Results from the handrinses collected in Term 3 indicate that the percent of pupils with any *E. coli* detected on their hands did not differ between the PSSD and GSD arms (79% and 78% in the PSSD and GSD arms, respectively; *p* = 0.73). The percent of pupils with high levels of *E coli* (≥100 CFU/hand) on their hands was 20% and 16% in the PSSD and GSD arms, respectively (*p* = 0.59).

In Term 1, 35 (35%) environmental swabs from FLTs in the PSSD arm tested positive for *E. coli* in Term 1, whereas 22 (14%) of the existing toilets in the GSD arm tested positive (*p* = 0.31). In Term 3, *E. coli* was detected in 21 (50%) of FLTs compared to 53 (59%) of new toilets in the GSD arm (*p* = 0.46). Over the three terms, the percentage of FLTs with detectable *E. coli* levels decreased (Term 1 = 42%, Term 2 = 32%, and Term 3 = 28%; *p* = 0.07).

**Figure 2:**
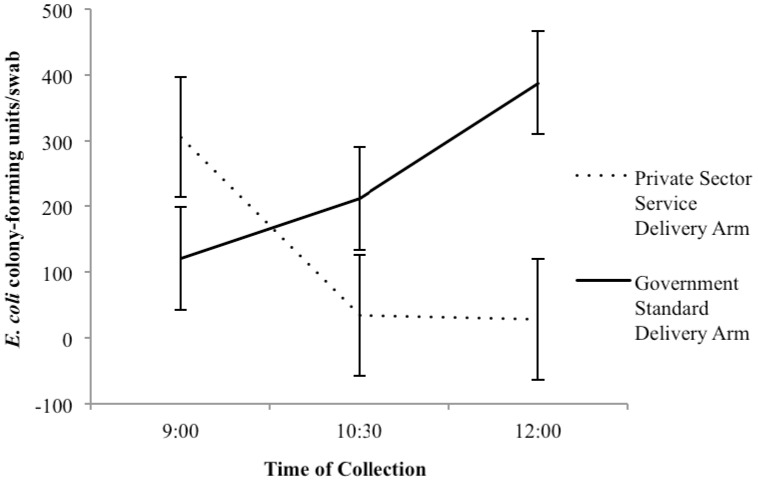
Geometric mean of samples collected throughout the day from toilets in both arms in Term 3.

In Term 3, *E. coli* was measured longitudinally over the course of one day. Figure 2 shows the geometric mean of samples collected throughout the day graphed against time of collection. Among FLTs, the mean number of *E. coli* colonies was highest at the beginning of the day and decreased throughout the day (*p* = 0.01). The new toilets in the GSD arm followed the opposite trend (*p* = 0.25), in which the mean number of *E. coli* colonies was lowest in the morning and increased as the day progressed.

### 3.6. Cost

The initial cost of the hardware and one year of field maintenance for private sector service delivery was USD 2053 (KES 210,000) per school. This was lower than the average cost of rehabilitating flush-type toilets in the GSD arm (USD 9306 (KES 922,638)) and the average cost of constructing new flush-type toilets (USD 11,489 (KES 1,169,668)), which did not include field maintenance support. Fewer visits (five visits) and less total personnel time (40 h) were used to install the FLTs than the toilets in the GSD arm (11 visits and 92 h). Additionally, the PSSD arm provided a comprehensive WASH training for teachers that cost USD 193 (KES 19,783). From the head teacher survey, schools in the PSSD arm reported having, on average, a larger budget for WASH facilities (USD 135 (KES 13,769)) than those in the GSD arm (USD 101 (KES 10,316)). The average intervention costs per school for both arms is shown in Table 3.

## 4. Discussion

We conducted a randomized trial to evaluate two modalities of sanitation delivery to schools in informal settlements. Our study demonstrates the potential for the private sector to deliver sanitation services to schools that is financially viable, sustainable, and scalable, and which could be achieved through public–private service delivery partnerships between the Ministry of Education and private sector enterprises. Feasibility of private sector service delivery of sanitation was defined as the ability to provide sustained sanitation services that were similar or superior to standard government sanitation approaches. Although there were no significant differences between the two approaches for some key indicators (exposure to *E. coli*, handwashing behaviors), toilet use as measured by infrared sensor was higher among FLTs (PSSD arm) compared to cistern-flush toilets (GSD arm), and toilet maintenance was better in the PSSD arm compared to the GSD arm. Additionally, the overall cost for private sector delivery and service of urine-diverting dry latrines was less expensive than the government standard of flush-type toilet provision. Further, flush-type toilets incur additional costs with water and sewage connections, which is not always sustainable in urban settings [3,4].

Although we observed no difference in daily toilet use between FLTs and cistern-flush toilets during the structured observation, installed PLUMS detected a 128% increase in use of the FLTs compared to cistern-flush toilets. This difference in measured outcomes could be because the structured observation of toilet use occurred across all schools, whereas PLUMs were deployed in only 12 schools during Term 3 and because structured observations were conducted on average 2 days during Term 3, whereas PLUMs were installed for an average of 21 days. Further, the presence of research staff has been shown to cause reactivity in participant behavior due to courtesy bias [33].

The rehabilitation of toilets in the GSD arm was met with several challenges, including limited access to sewer lines and piped water supply, unstable ground formation, blocked sewage drainage systems, vandalism, and eroded sewage pipes. Alternatively, the prefabricated toilets in the PSSD arm created one approach to sustainable solutions for sanitation through the daily, low-cost collection of waste, which is then converted into value-added byproducts, including organic fertilizer, insect-based animal feed, and renewable energy [34]. This closed-loop approach to sanitation has environmental benefits by preventing fecal pathogens from contaminating both groundwater and surface water. The urine-diverting dry sanitation model does not rely on water or sewage connections, nor does it need a large plot of land for construction, making it an ideal alternative for informal settlements, as previously studied in densely populated settlements in Kenya and Haiti [3,35]. However, some urine-diverting dry toilet designs are not always culturally acceptable, as noted by one school in the PSSD arm that was primarily composed of Muslim students. The school expressed more interest in flush-type toilets as opposed to FLTs, which do not provide a water connection or accommodate the diversion of anal cleansing water, which is necessary for ablution in accordance with the Islamic faith. Water is typically found in pit or cistern-flush latrine blocks for anal cleansing and/or flushing, whereas toilet paper or other solid materials are used for anal cleansing in urine-diverting dry toilets.

Toilets in both arms of the study were well maintained in Term 1 and Term 3, according to the maintenance score. The average numbers of toilet users remained steady, which may be a result of the sustained cleanliness of the toilets. Previous research has found an association between the level of cleanliness and the toilet usage [29,36,37]. The patterns in usage and cleanliness in this study corroborate that association, though more data are needed to support this finding.

The data indicate that over 75% of the pupils in both arms of the study presented with some *E. coli* on their hands, but there was no difference in contamination between the two study arms. The absence of observed handwashing with soap throughout the school year may explain this high prevalence of *E. coli* hand contamination. In Term 3, when these handrinses were collected, we found no differences in handwashing behaviors between the PSSD and GSD arms, and the majority of pupils did not wash their hands after a toilet event. Pupils’ hands could also have been re-contaminated based on their interactions with environmental surfaces, as previous studies have shown that various activities can increase hand contamination throughout the day [38]. However, the levels of *E. coli* measured on pupils’ hands cannot be directly related to risk of diarrheal diseases but rather serve as a proxy indicator of fecal bacteria [24]. While there is no established acceptable range for *E. coli* levels, ideally the pupils would not have any presence of fecal pathogens on their hands.

The decrease in handwashing with soap among pupils may be due to an ineffective hygiene promotion curriculum and the absence of daily provision of soap and water, which prevents handwashing behaviors from becoming habitualized. Despite having a budget allocated towards WASH facilities, schools in both arms of the trial struggled to provide sustained availability of soap, water, and anal cleansing materials. Schools should have the option of outsourcing all WASH-related consumables to the vendor (i.e., private sector enterprise) at an extra cost; the total cost of the PSSD including the provision of consumables would still be far cheaper than that of the government standard of flush-type toilets.

Despite the lack of statistical significance, we noted a trend in the environmental *E. coli* contamination in the swabs of toilets. The increase in *E. coli* throughout the day in the toilets of the GSD arm suggests that the surfaces are becoming contaminated with more usage, despite reported cleaning of the toilets. The difference in technologies between the two study arms would help support the conclusion that flush-type toilets result in the aerosolization of fecal contamination [39]. Further research would help confirm whether different toilet construction materials impact the survival rate of *E. coli* on surfaces.

There were several limitations to this study. First, we were constrained in our sample size based on the budget of the trial. Second, the difference in toilet materials (i.e., concrete versus tile) likely caused variances in *E. coli* recovery with swabs, which could bias the microbial data. Third, there was a nationwide teachers’ strike that closed all public schools for five weeks between September 2015 and October 2015. As a result, data collection was suspended in the 12 public schools enrolled in our study. In Term 1, three schools in the PSSD arm allocated the FLTs to girls only; therefore, the usage did not reflect that of the entire school. Lastly, and perhaps most importantly, the study was designed so that that installation of the interventions in all 20 schools would be completed by the beginning of the school year. However, construction in eight of the GSD schools was not complete until Term 2 and construction was never completed in two schools in the GSD arm. The delay in construction was, in part, because of unforeseen issues with access to a sewer connection, weak ground soil formation, and a high water table, representing a further challenge to the standard provision of these types of facilities. These delays created a challenge for us to conduct a head-to-head comparison of the performance of these toilets over the full school year. A follow-up study is planned to look at the sustained performance of these toilets over time.

In the case of the PLUM data, several limitations were observed. Firstly, the installation location of the PLUM varied between the arms. Each FLT is single-user, so one PLUM was positioned to detect use in one toilet; GSD toilets were constructed in blocks and it was not possible to position the PLUM over a single stance. As such, the PLUM was positioned to detect use between two and 10 toilets. Consequently, the measures were not equivalent and we therefore scaled the data by the median number of PLUM “clicks” per user, and then by the number of toilets observed per PLUM. There were considerably fewer observations available from GSD toilets compared to the FLTs.

Secondly, in previous PLUM studies, an event algorithm was derived from, and validated with, structured observations. This algorithm assumed gaps between users, as normally observed in households. In this study, this algorithm was not applied as the structured observations identified that many users were often present one after another. Consequently, the event algorithm in this study was necessarily simplified to be based on median “clicks” per user.

## 5. Conclusions

This study explored the feasibility of private sector sanitation provision for school children in informal settlements. We found few differences in observed use and exposure to fecal contamination between private sector service delivery and government standard delivery. However, sensor detected toilet use and toilet maintenance was superior in the PSSD arm compared to the GSD arm. Additionally, given the cost and time needed for installation, the private sector service delivery was approximately five times cheaper and a more expedient solution in the short term. A follow-up study will allow us to assess sustainability of the hardware and capture the life cycle costs of these two approaches. Our results support the provision of a private sector service delivery for school sanitation as an option in informal settlements as a complement to the existing provision options. A one-size-fits all approach to toilet designs is not sustainable in the informal settlements. Public–private service delivery partnerships could allow for the Ministry of Education to outsource sanitation responsibilities to private service providers to meet the demand for safely managed sanitation. Future research should explore the life cycle costs associated with the ability of schools to continue to pay for private sector services and sustained maintenance.

## Figures and Tables

**Table 1:** Comparison of Private Sector Service Delivery (PSSD) and Government Standard Delivery (GSD) intervention components.

Intervention Component	Private Sector Service Delivery (PSSD) (*n* = 10)	Government Standard Delivery (GSD) (*n* = 10)
Toilet type	Five pre-fabricated, container-based, urine-diverting dry toilets per school (10 schools)	One block of five cistern-flush toilets (9 schools) or one block of five ventilated improved pit (VIP) latrine (1 school)
Waste disposal	Service inclusive of daily collection of waste and off-site treatment	Connection to sewage and water (cistern-flush only)
Hygiene provision	One handwashing station (bucket with cover and tap) per school Hygiene promotion curriculum and teacher training	Between one and three handwashing stations (sink with multiple faucets), depending on number of students No hygiene promotion
Consumables	Waste cover material (sawdust)	None
Operations and maintenance support	Included	None

**Table 2:** Toilet characteristics and pupil hygiene behaviors for both trial arms.

Toilet Characteristics and Pupil Hygiene Behvaiors	Term 1			Term 3		
	Private Sector Service Delivery (PSSD) *n* = 10	Government Standard Delivery (GSD) *n* = 10	*p* ^§^	Private Sector Service Delivery (PSSD) *n* = 10	Government Standard Delivery (GSD) *n* = 10	*p* ^§^
*Sanitation*						
Toilet maintenance score ^1^	4.7 (0.8)	2.6 (1.4)	0.001	4.0 (0.3)	2.6 (1.2)	0.002
# of days in last school week toilets were cleaned	5 (0)	4.7 (0.04)	0.16	4.1 (1.8)	4.2 (1.3)	0.99
*Toilet Use*						
# toilets that appear to be available (i.e., unlocked)	86 (94%)	341 (84%)	0.37	39 (83%)	244 (88%)	0.46
# toilets that appear to be in use	80 (93%)	341 (100%)	0.28	39 (100%)	244 (100%)	-
(observed) % of pupils per school who used a toilet daily	12 (0.8)	14 (16)	0.1	15 (16.7)	20 (14.9)	0.86
*Handwashing*						
% pupils handwashing with soap and water	36.3 (35.3)	2.0 (5.7)	0.003	14.3 (23.5)	2.6 (5.7)	0.17
% pupils handwashing with water only	19.5 (22.2)	27.9 (11.4)	0.43	19.4 (15.7)	28.1 (24.0)	0.35
% pupils not handwashing	41.7 (23.4)	71.4 (15.1)	0.004	68.8 (21.0)	59.7 (28.8)	0.81
# of schools with water present at beginning of day	23 (95.8%)	13 (100%)	0.61	10 (100%)	12 (85.7%)	0.99
# schools with soap present at beginning of day	13 (54.2%)	0 (0%)	0.01	4 (40%)	2 (14.3%)	0.38

Data are presented as mean (SD) or n (%). ^1^ Toilet maintenance is composed of the following variables: presence of flies, presence of stagnant water/urine, odor, visible feces, cleaning supplies available, tissue paper available. ^§^ *p*-Value indicates statistical significance between PSSD and GSD arms at ∝ = 0.05.

**Table 3:** Average intervention costs per school for each trial arm.

Intervention Costs	Private Sector Service Delivery (PSSD) Arm (*n* = 10)	Government Standard Delivery (GSD) Arm (*n* = 10)
*Fixed hardware*		
Cost for toilet construction	USD 2053 (KES 210,000)	USD 10,518 (KES 1,079,764)
Number of visits for supervision of construction	5	11
Total personnel time used	40 h	92 h
*Curriculum*		
Cost for training	USD 194 (KES 19,783)	-
*Maintenance*		
Reported budget for facilities	USD 135 (KES 13,769)	USD 101 (KES 10,316)
Replacement parts	USD 5 (KES 500)	-
**Total Cost**	USD 2262 (KES 230,283)	USD 10,518 (KES 1,079,764)

## Supplementary Materials

The following are available online at www.mdpi.com/1660-4601/13/12/1189/s1, Figure S1: CONSORT 2010 Flow Diagram, Table S1: CONSORT 2010 checklist of information to include when reporting a randomised trial.
